# Health-related quality of life after gastrectomy, esophagectomy, and combined esophagogastrectomy for gastroesophageal junction adenocarcinoma

**DOI:** 10.1007/s10120-017-0761-2

**Published:** 2017-08-29

**Authors:** Joonas H. Kauppila, Cecilia Ringborg, Asif Johar, Jesper Lagergren, Pernilla Lagergren

**Affiliations:** 1 Upper Gastrointestinal Surgery, Department of Molecular Medicine and Surgery, Karolinska Institutet, Karolinska University Hospital, 17176 Stockholm, Sweden; 2 Cancer and Translational Medicine Research Unit, Medical Research Center Oulu, University of Oulu, Oulu University Hospital, Oulu, Finland; 3 Surgical Care Sciences, Department of Molecular Medicine and Surgery, Karolinska Institutet, Karolinska University Hospital, 17176 Stockholm, Sweden; 4 Division of Cancer Studies, King’s College London and Guy’s and St. Thomas’ NHS Foundation Trust, London, England, UK

**Keywords:** Esophageal neoplasm, Gastric cancer, Surgery, Symptoms, Functions

## Abstract

**Background:**

The postoperative health-related quality of life (HRQOL) outcomes in patients with gastroesophageal junction (GEJ) adenocarcinoma after gastrectomy and esophagectomy are unclear. The aim was to evaluate HRQOL outcomes 6 months after extended total gastrectomy, subtotal esophagectomy, and combined esophagogastrectomy.

**Methods:**

Patients who underwent surgery for GEJ adenocarcinoma of Siewert type 2 or 3 in 2001–2005 were identified from a nationwide Swedish prospective and population-based cohort. Three surgical strategies, i.e., gastrectomy, esophagectomy, or esophagogastrectomy, were analyzed in relationship to HRQOL measured at 6 months after surgery (main outcome). HRQOL was assessed using well-validated questionnaires for general (EORTC QLQ-C30) and esophageal cancer-specific (EORTC QLQ-OES18) symptoms. Mean score differences (MSD) and 95% confidence intervals (CI) were analyzed using ANCOVA and adjusted for age, sex, tumor stage, comorbidity, education level, hospital volume, and postoperative complications. MSDs > 10 were regarded as clinically relevant.

**Results:**

Among 176 patients with complete information on HRQOL and covariates, none of the MSDs for HRQOL among the three surgery groups were clinically and statistically significant. MSDs comparing esophagectomy and gastrectomy showed no major differences in global quality of life (MSD, +8, 95% CI, 0 to +16), physical function (MSD, +2, 95% CI, −5 to +9), pain (MSD, −3, 95% CI, −12 to +7), or reflux (MSD, +5, 95% CI, −4 to +14). Also, complication rates and 5-year survival rates were similar comparing esophagectomy and gastrectomy.

**Conclusions:**

Extended total gastrectomy, subtotal esophagectomy, and combined esophagogastrectomy seemed to yield similar 6-month postoperative HRQOL outcomes for patients with GEJ adenocarcinoma.

**Electronic supplementary material:**

The online version of this article (doi:10.1007/s10120-017-0761-2) contains supplementary material, which is available to authorized users.

## Introduction

The curative treatment of adenocarcinoma of the gastroesophageal junction (GEJ) typically includes surgical resection. Multimodal treatment and centralization of surgery have improved the 5-year survival rate [1, 2], but there is an ongoing debate about the optimal surgical approach for GEJ cancer [3]. None of the different surgical alternatives, i.e., total gastrectomy, subtotal esophagectomy, or a combination of these approaches, i.e., esophagogastrectomy, seems to offer superior oncological outcomes [4, 5]. However, it is important to also consider patients’ postoperative health-related quality of life (HRQOL). Poor HRQOL at 6 months after surgery for esophageal or gastroesophageal junction cancer is associated with increased long-term mortality [6, 7], as is poor HRQOL up to 10 years postoperatively [8–10]. HRQOL outcomes of extended total gastrectomy and subtotal esophagectomy have been studied only in a few, small, single-center studies with inherent problems with statistical power and selection bias. There is a need for larger studies based on unselected patients comparing extended gastrectomy, subtotal esophagectomy, and combined esophagogastrectomy, specifically examining patients with Siewert II and III GEJ cancer. Therefore, the aim of this study was to elucidate whether any of the three main alternative surgical procedures for GEJ cancer of Siewert II and III has a different impact on postoperative HRQOL at 6 months in a population-based and nationwide Swedish cohort study.

## Patients and methods

### Study design and data sources

We report here a nationwide Swedish, population-based, and prospective cohort study derived from the Swedish Esophageal and Cardia Cancer cohort (SECC), which has been described in detail elsewhere [10]. In brief, the SECC includes 90% of all patients who underwent surgery with curative intent for esophageal or GEJ cancer in Sweden during the period April 2, 2001 to December 31, 2005; these patients were followed up until February 2016. The prospectively collected information for the SECC included data on patient and tumor characteristics, surgical details, pre-defined complications occurring within 30 days of surgery, and the self-reported written HRQOL questionnaire at 6 months after surgery. Additionally, we obtained socioeconomic data from the Longitudinal Integration Database for Health Insurance and the Labor Market Studies (LISA) database, and information on comorbidities was obtained from the Swedish Patient Registry. The Registry of the Total Population provided highly accurate mortality data. All participating patients gave informed consent. The study was approved by the Regional Ethical Review Board in Stockholm, Sweden.

### Patients

Among all 616 patients enrolled in the SECC, 282 patients with a Siewert type II or III GEJ adenocarcinoma were eligible for the present study. Tumors of included patients had an epicenter up to 1 cm above or up to 5 cm below the GEJ [11]. Tumor staging was done according to the 6th edition of TNM classification of malignant tumors [12]. Location and tumor stage information was obtained from the pathology reports of the resected specimen.

### Exposure

Study exposure was any of the following three surgical procedures.

1. Total gastrectomy with resection of the distal esophagus through laparotomy and anastomosis just above the diaphragm; these patients were labeled the “gastrectomy group.”
2. Subtotal esophagectomy with resection of the proximal stomach through abdominal and thoracic incisions, and sometimes also neck incision, with a gastric pull-up reconstruction and an anastomosis in the upper chest or neck; these patients were labeled the “esophagectomy group.”
3. Combination of groups (1) and (2) with esophagogastrectomy and a long jejunal Roux-en-Y or colonic interposition with intrathoracic or neck anastomosis; these patients were labeled the “esophagogastrectomy group.”

### Outcomes

The primary outcome was HRQOL at 6 months after surgery. HRQOL was measured using well-established questionnaires developed and validated by the European Organisation for Research and Treatment of Cancer (EORTC) [13, 14]. The 30-item core questionnaire (QLQ-C30) has 9 multi-item scales measuring global quality of life, functions (physical, role, cognitive, emotional and social functioning) and symptoms (fatigue, pain, nausea and vomiting), and 6 single items measuring symptoms common among cancer patients in general (dyspnea, appetite loss, insomnia, constipation, diarrhea, financial impact) [13]. Symptoms common among esophageal cancer patients were measured with the supplemental module QLQ-OES18, which comprises 4 symptom scales (eating, reflux, esophageal pain, dysphagia) and 6 single items (cough, dry mouth, taste, choking, speech, and trouble swallowing saliva) [14]. Each item (on both questionnaires) has four response categories: “not at all,” “a little,” “quite a bit,” and “very much,” except for the global quality of life scale, which has seven response alternatives ranging from “very poor” to “excellent.”

Secondary outcomes were pre-defined complications within 30 days of surgery, as defined earlier [15], and 5-year overall mortality. The following complications were included: postoperative bleeding (>2 l or requiring reoperation), anastomotic leakage (symptomatic and verified clinically or radiologically), intraabdominal or intrathoracic abscesses (symptomatic or verified radiologically), sepsis (symptomatic with positive blood culture), pneumonia (symptomatic and verified radiologically), renal failure (need of dialysis), pulmonary embolism (verified radiologically), myocardial infarction (verified by electrocardiogram and enzymes), stroke (verified radiologically), and respiratory failure (need of intubation or mechanical ventilation).

### Statistical analysis

**Fig. 1:**
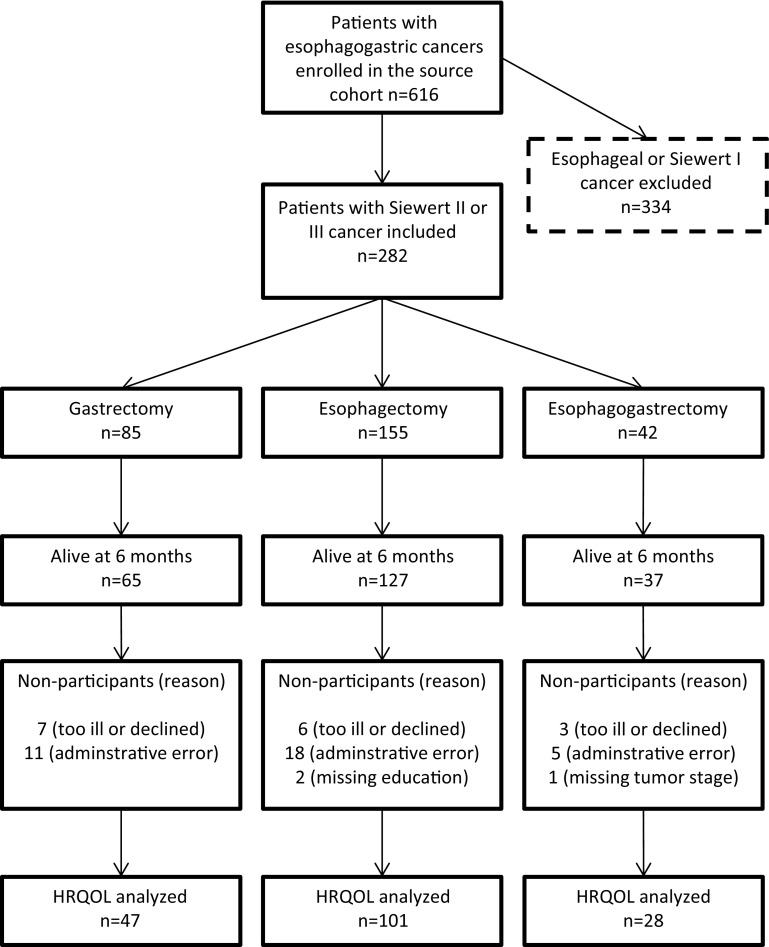
Flowchart of the study patients

The HRQOL questionnaire responses were transformed into scores between 0 and 100, and missing items were handled as recommended in the EORTC scoring manual [16]. Higher scores correspond to better HRQOL in the function scales and the global quality of life scale, whereas higher scores in symptom scales and items represent more problems. The main analysis included all the patients selected according to Fig. 1. Subgroup analysis for the primary and secondary outcomes was restricted to patients with Siewert type II GEJ cancer. Adjusted mean HRQOL scores for each surgical procedure were calculated with 95% confidence intervals (CI). Analysis of covariance (ANCOVA) was used to calculate adjusted mean score differences (MSD) with 95% CIs among the three surgery groups. Adjustment for confounding factors utilized a priori selected covariates potentially affecting HRQOL: age (categorized into <60 years, 60–74 years, or >74 years), sex (male or female), comorbidity (no or yes), tumor stage (0–I, II, III, or IV), education (9-year compulsory education, upper secondary education, or higher education), hospital volume (0–3, 4–9, or ≥10 operations per year), and pre-defined complications within 30 days of surgery (no or yes). To reduce errors resulting from multiple testing, we tested for statistical significance only when the MSDs were at least 10 between groups using the chi-square test. Such differences are considered clinically relevant and noticeable for the patient according to previous studies [17, 18].

To evaluate the association between surgical procedures and risk of pre-defined major complications, we used the multivariable logistic regression adjusted for the potential confounding variables already listed (except for complications). The relative risks were estimated using odds ratios (ORs) and 95% CIs.

Survival analysis used the Cox proportional hazards model while adjusting for the confounding variables listed here. The relative risks were expressed as hazard ratios (HRs) with 95% CIs.

The reference category in all statistical analyses was the gastrectomy group. The statistical software SAS V.9.4 (SAS Institute, Cary, NC, USA) was used for statistical analyses.

## Results

### Patients

Among 282 patients with GEJ adenocarcinoma in the cohort, 85 underwent gastrectomy, 155 underwent esophagectomy, and 42 had esophagogastrectomy. Among them, 229 patients (81%) survived for at least 6 months and 68 (24%) survived for 5 years or longer. Some characteristics of these 282 included patients are summarized in Table 1: 181 (64%) patients had Siewert type II cancer, 86 (31%) had Siewert type III cancer, and 15 (5%) had undetermined Siewert type II or III cancer. The patients in the gastrectomy group were more likely to have Siewert type III cancer and undergo surgery in a low-volume hospital compared to the esophagectomy group. Patients undergoing esophagogastrectomy were younger, had less comorbidity, fewer early-stage tumors, and were more likely to be treated in a high-volume hospital. Of the 229 patients surviving for 6 months, those 176 (77%) who responded to the HRQOL questionnaires and had complete data on all covariates were included in the HRQOL analyses. The distribution of nonparticipating patients was similar among the three surgery groups (21% for gastrectomy, 15% for esophagectomy, 17% for esophagogastrectomy). Characteristics of these patients, their tumors, and treatment were similar to that of all 282 participants (see Supplementary Table 1). A flowchart describing the study participants throughout the study is shown in Fig. 1. Only 8 patients (5%) received neoadjuvant therapy, and 11 patients (6%) received adjuvant therapy.

**Table 1:** Characteristics of the 282 patients who underwent surgery for gastroesophageal junction adenocarcinoma of Siewert type II or III

	Gastrectomy	Esophagectomy	Esophagogastrectomy	Total
	Number (%)	Number (%)	Number (%)	Number (%)
Total	85 (100)	155 (100)	42 (100)	282 (100)
Age (in years)				
<60	19 (22)	38 (25)	15 (36)	72 (26)
60–74	36 (42)	78 (50)	20 (48)	134 (48)
>74	30 (35)	39 (25)	7 (17)	76 (27)
Sex				
Male	69 (81)	128 (83)	33 (79)	230 (82)
Female	16 (19)	27 (17)	9 (21)	52 (18)
Education level				
9-year compulsory	47 (55)	72 (46)	13 (31)	132 (47)
Upper-secondary	27 (32)	53 (34)	21 (50)	101 (36)
Higher education	11 (13)	23 (15)	6 (14)	40 (14)
Missing	0 (0)	7 (5)	2 (5)	9 (3)
Comorbidity				
Yes	51 (60)	97 (63)	20 (48)	168 (60)
No	34 (40)	58 (37)	22 (52)	114 (40)
Siewert type				
II	37 (44)	122 (79)	22 (52)	181 (64)
III	47 (55)	20 (23)	19 (45)	86 (31)
Unclear II–III	1 (1)	13 (8)	1 (2)	15 (5)
Tumor stage				
0–I	19 (22)	29 (19)	2 (5)	50 (18)
II	27 (32)	38 (25)	15 (36)	80 (28)
III	25 (29)	66 (43)	18 (43)	109 (39)
IV	14 (16)	17 (11)	5 (12)	36 (13)
Missing	0 (0)	5 (3)	2 (5)	7 (2)
Hospital volume				
Low (0–3/year)	32 (38)	38 (25)	5 (12)	75 (27)
Mid (4–9/year)	30 (35)	38 (25)	12 (29)	80 (28)
High (≥10/year)	23 (27)	79 (51)	25 (60)	127 (45)

### Health-related quality of life 6 months after surgery

The adjusted HRQOL mean scores from the general cancer questionnaire (QLQ-C30) are presented in Table 2. Mean global quality of life scores were similar (MSD, +8, 95% CI, 0 to +16), as were physical function (MSD, +2, 95% CI, −5 to +9), and pain (MSD, −3 95% CI, −12 to +7) between the esophagectomy group and the gastrectomy group. Borderline MSDs were found for role function (MSD, +9, 95% CI, −3 to +20 and cognitive function (MSD, +10 [rounded up], 95% CI, +3 to +17) in the esophagectomy group compared to the gastrectomy group. Regarding general cancer symptoms, no clinically relevant differences were found between patients undergoing esophagectomy and the gastrectomy group (Table 2). However, patients in the esophagogastrectomy group reported clinically significantly more dyspnea than did the gastrectomy group, although this difference was not statistically significant (MSD, +12, 95% CI, −3 to +26).

**Table 2:** Health-related quality of life outcomes from the European Organization of Research and Treatment of Cancer (EORTC) QLQ-C30 questionnaire measures at 6 months after surgery for gastroesophageal junction adenocarcinoma of Siewert type II or III

	Gastrectomy *n* = 47	Esophagectomy *n* = 101	Esophagogastrectomy *n* = 28	Esophagectomy vs. gastrectomy	Esophagogastrectomy vs. gastrectomy
	Mean score (95% CI)	Mean score (95% CI)	Mean score (95% CI)	Mean score difference	Mean score difference
Global status					
Global quality of life	54 (47–62)	63 (57–69)	59 (49–69)	8	5
Functions					
Physical	75 (68–81)	77 (72–82)	72 (63–80)	2	3
Role	58 (47–68)	66 (58–75)	56 (42–70)	9	2
Emotional	69 (62–76)	73 (68–79)	71 (61–80)	4	2
Cognitive	75 (68–82)	85 (79–90)	84 (75–93)	10^a^	9
Social	69 (60–78)	72 (65–79)	61 (49–73)	3	−8
Symptoms					
Fatigue	49 (41–57)	41 (34–47)	50 (39–61)	−8	1
Nausea/vomiting	25 (18–33)	24 (18–30)	16 (6–25)	−1	−10^a^
Pain	27 (18–36)	24 (17–31)	21 (9–33)	−3	−6
Dyspnea	25 (15–35)	30 (22–38)	37 (24–50)	5	12*
Insomnia	26 (17–36)	25 (17–32)	20 (8–32)	−2	−6
Appetite loss	41 (29–52)	39 (30–48)	46 (31–61)	−2	5
Constipation	10 (3–17)	14 (9–20)	10 (1–19)	4	0
Diarrhea	31 (21–41)	27 (19–36)	22 (8–35)	−4	−9
Financial problems	13 (6–21)	14 (8–20)	11 (1–21)	1	−2

Data are presented as adjusted mean scores and 95% confidence intervals (CI) and mean score differences. Clinically significant differences (mean score difference of >10 scores) between comparison groups are bolded

^a^ Rounded value, not clinically significant

* *p* = 0.114, chi-squared test

The adjusted esophageal-specific HRQOL scores (QLQ-OES18) are presented in Table 3. No clinically relevant differences were found among any of the three surgery groups; patients who underwent esophagectomy reported fewer problems with dry mouth, but this difference was not statistically significant (MSD, −10, 95% CI, −20 to 0). Scores for dysphagia (MSD, −5, 95% CI, −14 to +4), esophageal pain (MSD, −2, 95% CI, −10 to +6), and reflux symptoms (MSD, 5, 95% CI, −4 to +14) were similar after esophagectomy compared to gastrectomy. A borderline clinical difference was found for coughing, wherein patients in the esophagectomy group reported higher scores than the gastrectomy group (MSD, +9, 95% CI, 0 to +18).

**Table 3:** Health-related quality of life outcomes from the European Organization of Research and Treatment of Cancer (EORTC) QLQ-OES18 questionnaire measures at 6 months after surgery for gastroesophageal junction adenocarcinoma of Siewert type II or III

	Gastrectomy *n* = 47	Esophagectomy *n* = 101	Esophagogastrectomy *n* = 28	Esophagectomy vs. gastrectomy	Esophagogastrectomy vs. gastrectomy
	Mean score (95% CI)	Mean score (95% CI)	Mean score (95% CI)	Mean score difference	Mean score difference
Symptoms					
Dysphagia	30 (21–38)	25 (18–32)	30 (19–41)	−5	0
Eating difficulties	35 (27–43)	36 (29–42)	32 (21–42)	1	−3
Reflux	20 (11–28)	25 (18–32)	11 (0–23)	5	−8
Esophageal pain	27 (19–35)	25 (18–31)	22 (11–32)	−2	−5
Trouble swallowing saliva	17 (9–24)	18 (12–24)	11 (1–21)	2	−5
Choked when swallowing	14 (6–22)	17 (11–23)	12 (2–22)	3	−2
Dry mouth	33 (23–43)	23 (15–31)	35 (22–48)	−10*	2
Trouble taste	28 (18–38)	21 (13–29)	28 (15–41)	−6	0
Trouble coughing	16 (8–25)	26 (19–33)	23 (12–35)	9	7
Trouble speaking	11 (4–17)	6 (0–11)	3 (−6 to 12)	−5	−8

Data are presented as adjusted mean scores and 95% confidence intervals (CI) and mean score differences. Clinically significant differences [mean score difference (MSD) >10 scores] between comparison groups are in bold font

* *p* = 0.052, chi-squared test

Subgroup analysis including only the 112 Siewert type II GEJ cancers suggested clinically relevantly better global quality of life (MSD, +17, 95% CI, +6 to +28), role (MSD, +11, 95% CI, −4 to +26), cognitive (MSD, +18, 95% CI, +8 to +28) and social function (MSD, +11, 95% CI, −2 to +25), as well as less fatigue (MSD, −14, 95% CI, −26 to −2) and less appetite loss (MSD, −11, 95% CI, −29 to +7) after esophagectomy compared to gastrectomy in the general cancer questionnaire (QLQ-C30) (Supplementary Table 2). The differences in global quality of life (*p* = 0.003), cognitive function (*p* < 0.001), and fatigue (*p* = 0.023) were also statistically significant. In the esophageal cancer-specific QLQ-OES18 questionnaire, esophagectomy was associated with clinically relevantly less dry mouth (MSD, −12, 95% CI, −27 to +2) and taste symptoms (MSD, −10, 95% CI, −26 to +5), but neither of these differences was statistically significant (Supplementary Table 2).

### Complications

In total, 97 (34%) of all 282 patients had at least one of the pre-defined complications within 30 days of surgery. Esophagogastrectomy had the lowest absolute 30-day complication rates (14%); the rate was higher for gastrectomy (40%) and for esophagectomy (37%). After adjustment for confounding variables, no difference in risk of complications was found between esophagectomy and gastrectomy (OR, 0.90, 95% CI, 0.50–1.63), but the esophagogastrectomy group had significantly lower complication rates compared to gastrectomy (OR, 0.31, 95% CI, 0.11–0.87).

In the subgroup analysis of patients with Siewert type II GEJ cancer, there was an indication of fewer complications following esophagectomy compared to gastrectomy, but no statistically significant association was found (HR, 0.43, 95% CI, 0.18–1.03) (Supplementary Table 3).

### Mortality

The absolute 5-year survival among all 282 patients was similar in the three surgery groups (24% for gastrectomy, 25% for esophagectomy, and 24% for esophagogastrectomy). There were no statistically significant differences in 5-year overall survival rates after adjustment for confounding variables: HR, 0.85, 95% CI, 0.62–1.17 for esophagectomy versus gastrectomy and HR, 0.87, 95% CI, 0.54 to 1.39 for esophagogastrectomy versus gastrectomy.

In the subgroup analysis restricted to patients with Siewert type II GEJ cancer, esophagectomy was associated with better survival compared to gastrectomy (HR, 0.62, 95% CI, 0.39–0.96) (Supplementary Table 3).

## Discussion

This study indicates that the choice between gastrectomy, esophagectomy, or esophagogastrectomy has no major influence on the HRQOL for GEJ cancer patients 6 months after surgery. The risks of complications and mortality were not clearly different when comparing the gastrectomy, esophagectomy, and esophagogastrectomy groups.

The strengths and the limitations of the study should be considered when interpreting the results. The prospective, nationwide population-based design counteracts information bias, selection bias, and recall bias. The nonparticipating patients might have poorer HRQOL compared to the patients participating in the study. However, the distribution of nonparticipating patients was similar among the surgery groups, and the compliance of the included patients was high (78%), making bias from this source less likely. The sample size was larger than in any other previous study on the topic. Neoadjuvant and adjuvant therapies became the standard of care only after the study period, which could be seen as a limitation. However, the low use of neoadjuvant and adjuvant treatment in the study population prevented confounding from these treatments. The well-validated EORTC questionnaires allow an accurate assessment of HRQOL in GEJ cancer patients [13, 14]. Finally, the results were adjusted for several potential confounding factors. The lack of baseline HRQOL data is a limitation. Thus, we could not adjust for potential preoperative differences in the HRQOL in the comparison groups. However, the characteristics of the patients were similar at baseline for the gastrectomy and esophagectomy groups. In addition, we included only Siewert II and III GEJ cancers, in which the choice of surgical procedure is dependent on the surgeon’s preference and experience rather than patient characteristics. In Siewert type II cancer, esophagectomy was more common, whereas gastrectomy was more common in type III patients. However, both procedures were used for both cancer types, confirming the rationale for including Siewert type II and III in the current study population. A larger proportion of esophagectomies were conducted in high-volume hospitals compared to gastrectomies, but this factor was adjusted for in the analyses. The small number of patients in the subgroup analyses, as well as patients who underwent esophagogastrectomy, limits the statistical power regarding these groups in particular. No *p* value correction was conducted, increasing the probability of false-positive findings (type I errors). Additionally, the patients who underwent esophagogastrectomy were younger and had less comorbidity at baseline. Therefore, the results of the subgroup analysis of Siewert type II, as well as those regarding the esophagogastrectomy group, must be interpreted with caution.

In previous studies, gastrectomy has been proposed to offer better short- and long-term postoperative HRQOL than esophagectomy, which is in conflict with the present study. However, the differences might be explained by methodological issues, including bias from selection and confounding in earlier studies. A study from the United States of 27 patients indicated better HRQOL after gastrectomy compared to esophagectomy more than 3 months after surgery [19]. Similarly, a study of 63 patients from the United Kingdom suggested better HRQOL outcomes following gastrectomy compared to esophagectomy at 6 months after surgery [20]. However, both these investigations were small, single-center studies with apparent problems with selection bias [19, 20]. Also, a recent German hospital-based study including 123 patients found a trend toward better HRQOL after gastrectomy compared to esophagectomy at least 24 months after surgery, which was mainly the result of fewer respiratory and reflux-related symptoms [21]. However, only 127 (36%) of the 357 eligible patients participated, suggesting a high risk of selection bias, and no adjustment for confounding variables was made [21]. Moreover, if the suggested threshold for clinical significance of 10 points on the quality of life scale would have been used in that study [17, 18], the only measure that would have favored gastrectomy would have been dyspnea [21].

The results of the present investigation suggest that the three surgical alternatives under study are comparable regarding HRQOL outcomes at 6 months after surgery for GEJ cancer. We used 6 months as the time point for the assessment because this is a window after the initial postoperative recovery period and before tumors tend to recur [7]. Poor HRQOL at 6 months has also been shown associated with poor survival, as is poor HRQOL in the long term [6–10]. Physical function and pain, which should be the aspects most affected by the trauma caused by extensive surgery, were only minimally different between the groups. It has been proposed previously that the perception of HRQOL after surgery is not affected to a large extent by major cancer surgery because the patients are happy about surviving [24]. It has been shown that HRQOL is affected more and continues to deteriorate postoperatively in patients with comorbidities, whereas the majority of patients recover well after surgery [25]. Surgical complications also predict poor HRQOL [15], stressing the relevance of adjusting for complications to examine the role of the surgical approach per se. According to a recent interview study, patients want more information on recovery and quality of life after surgery [26]. Taken together, patients should be informed about the expected postoperative quality of life following surgery.

The subgroup analysis restricted to Siewert type II GEJ cancers only suggested that esophagectomy might be associated with better global HRQOL, cognitive function, as well as less fatigue, compared to gastrectomy, which is probably a result of the small number of patients in the analysis. A randomized controlled trial from Japan suggested aggravated weight loss, symptoms such as reduced meal volume, more pain, and more dyspnea, as well as more respiratory problems, in the long term after surgery, including a thoracotomy (left thoracoabdominal approach) for gastric cancer, compared to a non-thoracotomy procedure (transhiatal abdominal approach) [22]. Greater surgical trauma might also associate with worse symptoms in a meta-analysis comparing HRQOL outcomes after minimally invasive and open esophagectomy for cancer, at least in the short term [23]. However, the study populations and surgical approaches in these two studies are not entirely comparable with the present study. Taken together, the results of the subgroup analysis do not fit in the hypothesis that less surgical trauma results in better HRQOL, but do indicate a need for further studies comparing HRQOL outcomes between surgical approaches in Siewert type II GEJ cancer.

The 30-day complication rates were similar between the gastrectomy (40%) and esophagectomy (37%) groups, whereas the lower complication rate in the esophagogastrectomy group is most likely explained by selection of more fit patients for such extensive surgery. As in this cohort, earlier comparative studies between gastrectomy and esophagectomy for GEJ cancer have reported complication rates between 30% and 54% [27–29]. There was no 5-year survival benefit for any of the operative techniques studied in the main analysis, which is well in line with most previous research [30–32], and stresses the relevance of studying patient-reported outcomes. The observed association between esophagectomy and better 5-year survival in the subgroup analysis of Siewert type II GEJ cancer needs further research in better powered studies.

In conclusion, the 6-month postoperative HRQOL seems to be similar comparing the surgical approaches of extended total gastrectomy, subtotal esophagectomy, and esophagogastrectomy in patients with GEJ adenocarcinoma of Siewert type II and III. These results indicate that the choice of surgical approach could be based on the preference of the surgeon.

## Electronic supplementary material

Below is the link to the electronic supplementary material.

Supplementary material 1 (DOCX 26 kb)
