# Supplementary material for: Health-related quality of life after gastrectomy, esophagectomy, and combined esophagogastrectomy for gastroesophageal junction adenocarcinoma
Source: Gastric Cancer. 2017 Aug 29;21(3):533–41. doi: 10.1007/s10120-017-0761-2 (PMC5906505; doi:10.1007/s10120-017-0761-2)
Supplement: Supplementary file 1 — Supplementary material 1 (DOCX 26 kb) [file 10120_2017_761_MOESM1_ESM.docx]

**Supplementary data for original article:**

**Health-related quality of life after gastrectomy, esophagectomy and combined esophagogastrectomy for gastroesophageal junction adenocarcinoma**

**Journal name:** Gastric Cancer

**Authors:** Joonas H Kauppila,^1,2^ MD, PhD, Cecilia Ringborg,^3^ RN, MSc, Asif Johar,^3^ MSc, Jesper Lagergren,^1,4^ MD, PhD, and Pernilla Lagergren,^3^ RN, PhD.

**Affiliations:** ^1^Upper Gastrointestinal Surgery, Department of Molecular Medicine and Surgery, Karolinska Institutet, Karolinska University Hospital, 17176 Stockholm, Sweden; ^2^Cancer and Translational Medicine Research Unit, Medical Research Center Oulu, University of Oulu, Oulu University Hospital, Oulu, Finland; ^3^Surgical Care Sciences, Department of Molecular Medicine and Surgery, Karolinska Institutet, Karolinska University Hospital, 17176 Stockholm, Sweden; ^4^Division of Cancer Studies, King’s College London and Guy's and St Thomas' NHS Foundation Trust, London, England.

**Corresponding author:**

Joonas H Kauppila, Upper Gastrointestinal Surgery, Department of Molecular Medicine and Surgery, Karolinska Institutet, Karolinska University Hospital, 17176 Stockholm, Sweden Email: [joonas.kauppila@ki.se](mailto:joonas.kauppila@ki.se).

**Supplementary Table 1.** Characteristics of 176 patients who underwent surgery for gastroesophageal junction adenocarcinoma of Siewert type II or III and responded to the quality of life questionnaires 6 months after surgery.

|  | **Gastrectomy** | **Esophagectomy** | **Esophagogastrectomy** | **Total** |
| --- | --- | --- | --- | --- |
|  | Number (%) | Number (%) | Number (%) | Number (%) |
| **Total** | 47 (100) | 101 (100) | 28 (100) | 176 (100) |
|  |  |  |  |  |
| **Age (in years)** |  |  |  |  |
| <60 | 13 (28) | 25 (25) | 11 (39) | 49 (28) |
| 60-74 | 20 (43) | 51 (50) | 12 (43) | 83 (47) |
| >74 | 14 (30) | 25 (25) | 5 (18) | 44 (25) |
|  |  |  |  |  |
| **Sex** |  |  |  |  |
| Male | 36 (77) | 82 (81) | 23 (82) | 141 (80) |
| Female | 11 (23) | 19 (19) | 5 (18) | 35 (20) |
|  |  |  |  |  |
| **Hospital volume** |  |  |  |  |
| Low (0-3/year) | 12 (26) | 27 (27) | 3 (11) | 42 (29) |
| Mid (4-9/year) | 21 (45) | 27 (37) | 8 (29) | 56 (32) |
| High (≥10/year) | 14 (30) | 47 (47) | 17 (61) | 78 (44) |
|  |  |  |  |  |
| **Comorbidity** |  |  |  |  |
| Yes | 28 (60) | 59 (58) | 12 (43) | 99 (56) |
| No | 19 (40) | 42 (42) | 16 (57) | 77 (44) |
|  |  |  |  |  |
| **Siewert type** |  |  |  |  |
| II | 18 (38) | 78 (77) | 16 (57) | 112 (64) |
| III | 29 (62) | 14 (14) | 11 (39) | 54 (31) |
| Unclear II-III | 0 (0) | 9 (9) | 1 (4) | 10 (6) |
|  |  |  |  |  |
| **Tumor stage** |  |  |  |  |
| 0-I | 12 (26) | 21 (21) | 2 (7) | 35 (20) |
| II | 12 (26) | 24 (24) | 11 (39) | 47 (27) |
| III | 19 (40) | 46 (46) | 11 (39) | 76 (43) |
| IV | 4 (9) | 10 (10) | 4 (14) | 18 (10) |
|  |  |  |  |  |
| **Education level** |  |  |  |  |
| 9-year compulsory | 25 (53) | 47 (47) | 9 (32) | 81 (46) |
| Upper-secondary | 15 (32) | 35 (35) | 14 (50) | 64 (36) |
| Higher education | 7 (15) | 19 (19) | 5 (18) | 31 (18) |
|  |  |  |  |  |
| **Complications** |  |  |  |  |
| No | 34 (72) | 70 (69) | 25 (89) | 129 (73) |
| Yes | 13 (28) | 31 (31) | 3 (11) | 47 (27) |

**Supplementary Table 2.** Health-related quality of life outcomes from the European Organization of Research and Treatment of Cancer (EORTC) QLQ-C30 and QLQ-OES18 questionnaires measures at 6 months after surgery for gastroesophageal junction adenocarcinoma of Siewert type II. Data are presented as mean scores and 95% confidence intervals (CI) and mean score differences. The estimates were adjusted for age, sex, education, comorbidity, tumor stage, hospital volume and complications. Clinically significant differences (mean score difference of >10 scores) between comparison groups are bolded.

|  | | **Gastrectomy**  **n=18** | **Esophagectomy**  **n=78** | | | **Esophago-gastrectomy**  **n=16** | | **Esophagectomy vs gastrectomy** | **Esophago-gastrectomy vs gastrectomy** |
| --- | --- | --- | --- | --- | --- | --- | --- | --- | --- |
|  | | Mean score (95% CI) | Mean score  (95% CI) | | | Mean score (95% CI) | | Mean score difference | Mean score difference |
| **EORTC QLQ-C30** | | | | | | | | | |
| **Global status** | |  |  | | |  | |  |  |
| Global quality of life | | 44 (33 to 55) | 60 (54 to 67) | | | 46 (33 to 58) | | **16**^1^ | 2 |
| **Functions** | |  |  | | |  | |  |  |
| Physical | | 71 (61 to 81) | 76 (70 to 82) | | | 64 (52 to 75) | | 5 | -7 |
| Role | | 52 (37 to 67) | 63 (54 to 72) | | | 40 (23 to 57) | | **11**¶ | **-12**¶ |
| Emotional | | 70 (59 to 80) | 71 (64 to 77) | | | 64 (52 to 76) | | 1 | -6 |
| Cognitive | | 68 (58 to 78) | 86 (80 to 92) | | | 79 (67 to 90) | | **18^2^** | **11**¶ |
| Social | | 59 (45 to 72) | 70 (62 to 78) | | | 47 (32 to 62) | | **11**¶ | **-12**¶ |
| **Symptoms** | |  |  | | |  | |  |  |
| Fatigue | | 58 (46 to 70) | 44 (36 to 51) | | | 64 (50 to 78) | | **-14^3^** | 6 |
| Nausea/ vomiting | | 33 (22 to 45) | 26 (19 to 33) | | | 26 (13 to 40) | | -7 | **-**7 |
| Pain | | 28 (15 to 41) | 22 (14 to 30) | | | 26 (11 to 41) | | -5 | -2 |
| Dyspnea | | 36 (21 to 52) | 34 (25 to 43) | | | 41 (24 to 59) | | -2 | 5 |
| Insomnia | | 24 (10 to 38) | 25 (16 to 33) | | | 19 (3 to 35) | | 1 | -5 |
| Appetite loss | | 51 (33 to 69) | 40 (29 to 50) | | | 65 (44 to 85) | | **-11**¶ | **14**¶ |
| Constipation | | 11 (1 to 22) | 16 (9 to 22) | | | 13 (1 to 24) | | 4 | 1 |
| Diarrhea | | 32 (16 to 49) | 33 (23 to 43) | | | 37 (18 to 56) | | 1 | 4 |
| Financial problems | | 16 (4 to 28) | 15 (8 to 22) | | | 14 (0 to 27) | | -1 | -2 |
|  | | | | | | | | | |
| **EORTC QLQ-OES18** | | | | | | | | | |
|  | **Gastrectomy**  **n=18** | | | **Esophagectomy**  **n=78** | **Esophago-gastrectomy**  **n=16** | | **Esophagectomy vs gastrectomy** | | **Esophago-gastrectomy vs gastrectomy** |
|  | Mean score (95% CI) | | | Mean score  (95% CI) | Mean score (95% CI) | | Mean score difference | | Mean score difference |
| Dysphagia | 26 (13 to 39) | | | 23 (15 to 31) | 31 (17 to 46) | | -3 | | 5 |
| Eating difficulties | 42 (30 to 54) | | | 37 (30 to 44) | 41 (27 to 55) | | -5 | | -1 |
| Reflux | 14 (1 to 28) | | | 24 (15 to 31) | 10 (-5 to 25) | | 9 | | -5 |
| Esophageal pain | 31 (18 to 44) | | | 23 (15 to 30) | 23 (8 to 37) | | -8 | | -8 |
| Trouble swallowing saliva | 11 (-1 to 23) | | | 16 (9 to 23) | 21 (8 to 35) | | 5 | | **10**¶ |
| Chocked when swallowing | 9 (-3 to 20) | | | 16 (9 to 23) | 19 (6 to 32) | | 7 | | 10† |
| Dry mouth | 34 (20 to 49) | | | 22 (13 to 31) | 41 (25 to 58) | | **-12**¶ | | 7 |
| Trouble taste | 33 (17 to 48) | | | 22 (13 to 31) | 39 (21 to 56) | | **-10**¶ | | 6 |
| Trouble coughing | 20 (5 to 35) | | | 26 (17 to 35) | 24 (7 to 41) | | 6 | | 4 |
| Trouble speaking | 9 (-1 to 19) | | | 6 (0 to 12) | 0 (-12 to 11) | | -3 | | -9 |

^1^p=0.003; ^2^p=0.0004; ^3^ p=0.023; ¶, not significant; † rounded value, not clinically significant. .

**Supplementary table 3.** The risk of complications and 5-year overall mortality in the main model and the subgroup analysis of Siewert II GEJ cancers by surgical approach. Complications are analysed using logistic regression, presented as odds ratios (ORs) and 95% confidence intervals (CIs) and 5-year mortality using Cox proportional-hazards regression analysis, presented as hazard ratios (HRs) and 95% confidence intervals (CIs).

|  | **Patients** | **Gastrectomy** | **Esophagectomy** | **Esophago-gastrectomy** |
| --- | --- | --- | --- | --- |
| **Complications** |  |  |  |  |
| **Model** | Number | OR (95% CI) | OR (95% CI) | OR (95% CI) |
| All patients* | 273† | 1 (Ref.) | 0.90 (0.50-1.63) | 0.31 (0.11-0.87) |
| Siewert type II* | 173†† | 1 (Ref.) | 0.43 (0.18-1.03) | 0.04 (0.00-0.33) |
|  |  |  |  |  |
| **5-year mortality** |  |  |  |  |
| **Model** | Number | HR (95% CI) | HR (95% CI) | HR (95% CI) |
| All patients** | 273† | 1 (Ref.) | 0.85 (0.62-1.17) | 0.87 (0.54-1.39) |
| Siewert II** | 173†† | 1 (Ref.) | 0.62 (0.39-0.96) | 0.59 (0.30-1.17) |

*Adjusted for age, sex, education, comorbidity, tumor stage and hospital volume.

**Adjusted for age, sex, education, comorbidity, tumor stage, hospital volume and complications

†Nine patients were excluded because of missing data.

†† Eight patients were excluded because of missing data.
